# Megf10 deficiency impairs skeletal muscle stem cell migration and muscle regeneration

**DOI:** 10.1002/2211-5463.13031

**Published:** 2020-11-26

**Authors:** Chengcheng Li, Dorianmarie Vargas‐Franco, Madhurima Saha, Rachel M. Davis, Kelsey A. Manko, Isabelle Draper, Christina A. Pacak, Peter B. Kang

**Affiliations:** ^1^ Division of Pediatric Neurology Department of Pediatrics University of Florida College of Medicine Gainesville FL USA; ^2^ Molecular Cardiology Research Institute Department of Medicine Tufts Medical Center Boston MA USA; ^3^ Department of Pediatrics University of Florida College of Medicine Gainesville FL USA; ^4^ Department of Molecular Genetics & Microbiology and Department of Neurology University of Florida College of Medicine Gainesville FL USA; ^5^ Genetics Institute and Myology Institute University of Florida Gainesville FL USA; ^6^ Present address: Washington University in St. Louis 660 South Euclid Avenue St. Louis MO 63110 USA; ^7^ Present address: Lacerta Therapeutics 12085 Research Drive, Suite #46 Alachua FL 32615 USA; ^8^ Present address: DeBusk College of Osteopathic Medicine Lincoln Memorial University Harrogate TN 37752 USA; ^9^ Present address: University of Central Florida College of Medicine 6850 Lake Nona Boulevard Orlando FL USA

**Keywords:** MEGF10 myopathy, satellite cells, skeletal muscle regeneration

## Abstract

Biallelic loss‐of‐function *MEGF10* mutations lead to MEGF10 myopathy, also known as early onset myopathy with areflexia, respiratory distress, and dysphagia (EMARDD). MEGF10 is expressed in muscle satellite cells, but the contribution of satellite cell dysfunction to MEGF10 myopathy is unclear. Myofibers and satellite cells were isolated and examined from *Megf10^−/−^* and wild‐type mice. A separate set of mice underwent repeated intramuscular barium chloride injections. *Megf10^−/−^* muscle satellite cells showed reduced proliferation and migration, while *Megf10^−/−^* mouse skeletal muscles showed impaired regeneration. Megf10 deficiency is associated with impaired muscle regeneration, due in part to defects in satellite cell function. Efforts to rescue Megf10 deficiency will have therapeutic implications for MEGF10 myopathy and other inherited muscle diseases involving impaired muscle regeneration.

## Graphical abstract

The exact disease mechanism of MEGF10 deficiency, which causes a rare inherited myopathy, remains unknown. In mice, Megf10 deficiency is associated with slower proliferation and migration of satellite cells, as well as impaired skeletal muscle regeneration after skeletal muscle injury experiments. These studies demonstrate that Megf10 makes important contributions to the function of satellite cells in muscle regeneration.

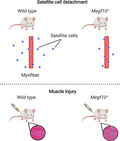

**Abbreviations**
AMP: adult muscle precursor
ANOVA: analysis of variance
bHLH: basic helix‐loop‐helix
Drpr: Draper
EDL: extensor digitorum longus
EMARDD, early onset myopathy: areflexia, respiratory distress, and dysphagia
IACUC: institutional animal care and use committee
IF: immunofluorescence
MEGF10 (human) or Megf10 (mouse): multiple EGF‐like domains 10
MRF: myogenic regulatory factor
PBS: phosphate‐buffered saline
SEM: standard error of the mean
SSRI: selective serotonin reuptake inhibitor
TA: tibialis anterior

MEGF10 is an orphan receptor that is expressed in developing myoblasts and muscle satellite cells. Biallelic loss‐of‐function mutations in the encoding gene *MEGF10* cause a recessive congenital muscle disease called MEGF10 myopathy [1, 2, 3, 4, 5, 6, 7]; the classic form of this disease is also known as early onset myopathy with areflexia, respiratory distress, and dysphagia (EMARDD) [8]. Minicores have been seen on muscle biopsy in several cases of MEGF10 myopathy [2, 5], and an adult onset form of this disease has also been described [4, 5]. The molecular mechanism of disease in MEGF10 myopathy/EMARDD involves impaired tyrosine phosphorylation [9] and impaired interactions between MEGF10 and the Notch pathway [10, 11], whereas the cellular mechanism of disease appears to involve potential defects in myogenesis, particularly myoblast proliferation [10] and migration [11], consistent with the congenital onset of disease in the classic EMARDD phenotype.

Satellite cells are muscle stem cells that are located between the sarcolemma and the basal lamina of muscle fibers and were first described in frogs [12]. Embryologically, they originate from the dermomyotome cell population [13]. Satellite cells undergo asymmetric division, yielding both a self‐perpetuating cell population and cells that are destined to fuse with adjacent muscle fibers to assist with muscle growth and regeneration [14]. In the settings of muscle injury and chronic muscle disease, satellite cells help resuscitate injured and regenerating muscle fibers by fusing with them [15]. The regulation of satellite cell states, including quiescence, activation, proliferation, and differentiation/fusion, is complex, with many details described but many questions still unanswered [16]. In the context of MEGF10, prior work has shown that satellite cell dysfunction contributes to the pathogenesis of MEGF10 myopathy and that Megf10 is expressed in quiescent and activated murine satellite cells [10]. Dll1 [17], a Notch ligand, and myogenin [18] have been shown to be positive regulators of Megf10 expression, whereas the combination of an *Rbpj* conditional mutation in Pax3+ myogenic precursors combined with mutant *MyoD* downregulate Megf10 expression [17]. However, it is not yet clear whether Megf10 deficiency has an impact on key cellular processes such as satellite cell migration, along with the larger biological process of muscle regeneration. Experiments on a mouse model of MEGF10 myopathy as well as satellite cells isolated from these mice will help answer these questions.

There are several methods used to replicate muscle injury in mouse models [19]. For the current study, we selected chemical injury with barium chloride due to its reliability and low toxicity [20]. A prior study from our group showed that muscle injury induced by intramuscular barium chloride injections yielded subtle signs of delayed regeneration in *Megf10^−/−^* mice compared to wild‐type mice [11]. However, repeated intramuscular injections to deplete the satellite cell pool have not previously been performed [21].

A better understanding of the contribution of MEGF10 to satellite cell function and muscle regeneration, along with the effects of MEGF10 deficiency on these important processes, could help identify novel targets for therapeutic strategies for MEGF10 myopathy. If such therapeutic strategies specifically augment satellite cell function in skeletal muscle, they could also be applied to a range of muscle diseases associated with impaired regeneration, including the muscular dystrophies. The aims of the current study are to examine patterns of satellite cell detachment from myofibers, expression patterns of MyoD in these satellite cells, and skeletal muscle regenerative capacity in a mouse model of MEGF10 myopathy.

## Materials and methods

### Mouse strains

All animal studies were performed under the auspices of a protocol approved by the Institutional Animal Care and Use Committee (IACUC) at the University of Florida. *Megf10^+/‐^* mice with a C57BL/6 background were generous gifts from Jeremy Kay at Duke University and Joshua Sanes at Harvard University, and were bred to yield *Megf10^−/−^* mice. Genotypes were verified as previously described, and Megf10 deficiency has previously been confirmed in this strain [11, 22]. Wild‐type C57BL/6J mice were obtained from Animal Care Services at the University of Florida. Skeletal muscle specimens for histological analysis and cell extractions were dissected *ex vivo* immediately after IACUC‐approved euthanasia procedures.

### Single myofiber isolation and culture, with satellite cell detachment

Individual myofibers were isolated from the extensor digitorum longus (EDL) muscles of 6 *Megf10^−/−^* and 5 wild‐type mice according to a standard protocol [23]. EDL is a standard muscle from which to isolate individual myofibers from mice for satellite cell analysis [23]. Ten to fifteen live myofibers were dissected from each EDL muscle and then cultured individually in glass chamber slides (ibidi GmbH, Martinsried, Germany) coated with Matrigel (Corning^®^) with serum‐rich medium (20% fetal bovine serum). The satellite cells observed to be detached from each myofiber were manually counted at 24, 48, and 72 h timepoints to capture detachment and migration prior to differentiation [24].

### Immunofluorescence (IF) of myofibers and satellite cells

At 72 h, the myofibers and satellite cells in glass chamber slides were washed with phosphate‐buffered saline (PBS), fixed in prewarmed 4% paraformaldehyde, incubated in 1% glycine in PBS quenching solution to minimize background staining, then permeabilized with 0.1% Triton X‐100 in PBS. After blocking with 5–10% fetal bovine serum in PBS, the myofibers and satellite cells were incubated with primary antibodies to Pax7 (Developmental Studies Hybridoma Bank) and MyoD (Developmental Studies Hybridoma Bank and Santa Cruz), then with secondary Alexa‐568 or Alexa‐488 conjugated goat anti‐rabbit antibodies (Life Technologies) or secondary Alexa‐568 anti‐mouse antibodies (Life Technologies). The nuclei were counterstained with DAPI. Pax7+ and MyoD‐+ satellite cells were counted, and the shortest distance between the center of each satellite cell to the edge of the myofiber was measured using ImageJ [25].

### IF of satellite cells

Myofibers were cultured on cover slips in culture dishes and maintained in proliferation media consisting of 20% fetal bovine serum, 5% chick embryo extract, and 2% penicillin/streptomycin in DMEM (high glucose). At 72 h, the myofibers were washed away, leaving the activated satellite cells on the culture dish. IF was performed on the satellite cells as described above for MyoD, accompanied by nuclear DAPI counterstaining.

### Barium chloride injury

Muscle injury was modeled in 2‐month‐old mice of both strains via injection of 50 μL 1.2% barium chloride in sterile water into the right tibialis anterior, with injection of sterile water into the left tibialis anterior as a control. Tibialis anterior is a standard muscle used for such muscle injury experiments in mice [26]. To deplete the satellite cell pool, the injections were performed a total of three times [21] starting at age 2 months, with gaps of 21–28 days between injections to permit complete recovery between injections [27]. Twenty‐one days after the third injection, the mice were euthanized and bilateral tibialis anterior muscles were harvested. The tibialis anterior muscles were snap‐frozen for hematoxylin and eosin staining.

### Hematoxylin and eosin histological analyses

Tibialis anterior muscles were dissected immediately *ex vivo* from the two mouse strains noted above and then snap‐frozen in 2‐methylbutane cooled with liquid nitrogen. The muscle samples were sectioned at 7µm in a cryostat and transferred to superfrost microscope slides. For hematoxylin and eosin staining, the sections were incubated for 5 minutes in hematoxylin (Leica Biosystems); rinsed with cold tap water; immersed twice in eosin Y; washed consecutively with 80, 90, and 100% ethanol; and incubated three times for 2 minutes in xylene. Sections were also stained with fibronectin using standard methods [28]; fibronectin quantification was performed using ImageJ [25]. The sections were mounted with Cytoseal 60 (Richard Allan Scientific) and visualized on an Olympus BX51 upright light microscope.

### Statistical analysis

Unpaired *t*‐tests were used to compare Megf10*^−/−^* and wild‐type groups for the various experiments described above. Means +/‐ standard error of the mean (SEM) were calculated. One‐way analysis of variance (ANOVA) was used to analyze the fibronectin quantification. GraphPad Prism (GraphPad Software, San Diego, California) was used for statistical analyses.

## Results

### ***Diminished detachment of Megf10*** *^−/−^* ***satellite cells from myofibers compared to wild‐type cells***

**Fig. 1:**
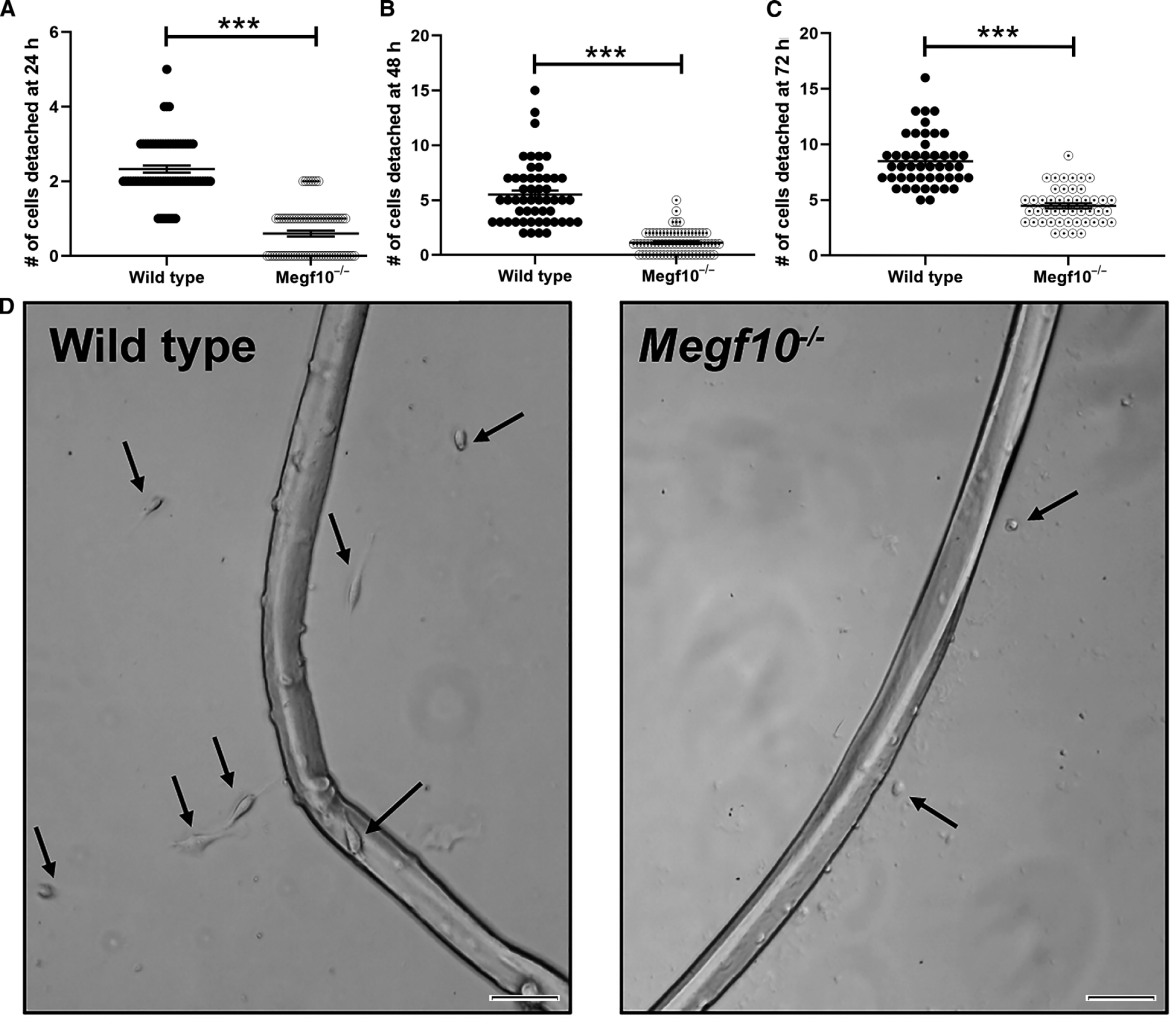
(A) Single intact live myofibers were isolated from wild‐type and *Megf10^−/−^* mice and cultured in serum‐rich medium in glass chamber slides. Detached satellite cells were counted for each myofiber at (A) 24, (B) 48, and (C) 72 h timepoints. Each dot represents the satellite cell count for one myofiber. The number of myofibers evaluated were: (A) wild‐type, *n* = 70 myofibers; *Megf10^−/−^*, *n* = 70 myofibers; (B) wild‐type, *n* = 52 myofibers; *Megf10^−/−^*, *n* = 70 myofibers; (C) wild‐type, *n* = 47 myofibers; *Megf10^−/−^*, *n* = 47 myofibers. Comparisons between wild‐type and *Megf10^−/−^* myofiber counts were performed at each time point by unpaired *t*‐tests; ***, *P* < 0.001. (D) Representative images are shown at 72 h. Arrows indicate satellite cells that have detached and migrated from the isolated myofibers. Scale bar, 50 μm.

Manual counts showed steadily increasing numbers of satellite cells that had visibly detached from individual myofibers at 24 h (Fig. 1A), 48 h (Fig. 1B), and 72 h (Fig. 1C) of culture. Representative images are shown at 72 hours (Fig. 1D). At each time point, fewer satellite cells were detached from *Megf10^−/−^* live myofibers than from wild‐type live myofibers, indicating that Megf10 deficiency is associated with impaired satellite cell activation and migration.

### ***Deficiency in MyoD expression of Megf10*** *^−/−^* ***satellite cells***

**Fig. 2:**
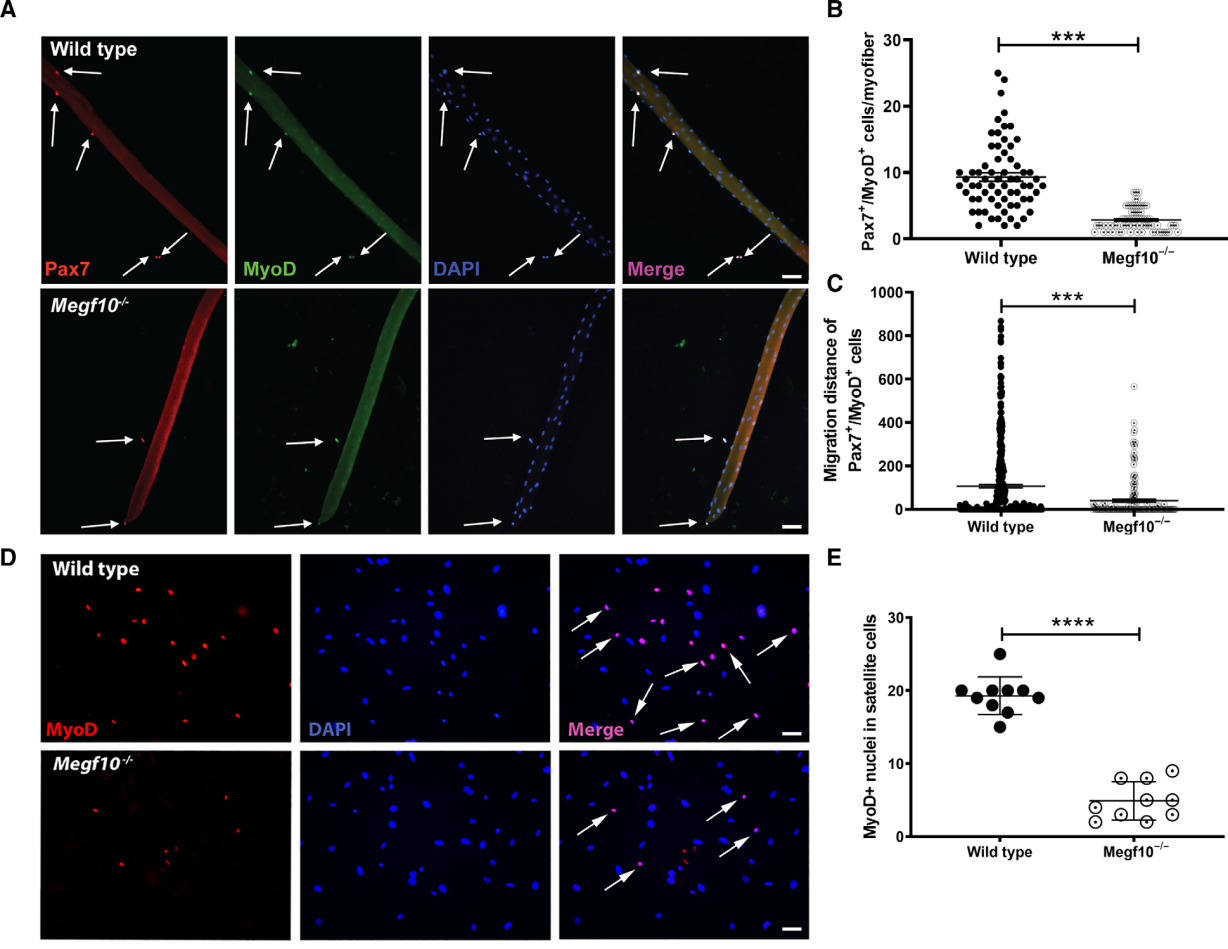
(A) Representative individual and merged images of satellite cells stained with anti‐Pax7 (red), anti‐MyoD (green), and DAPI at 72 h of myofiber culture. Arrows indicate satellite cells expressing both Pax7 and MyoD. Scale bar = 50 μm. (B) Scatter plot showing numbers of Pax7+/MyoD+ satellite cells associated with individual myofibers at 72 h. Each dot represents the cell count from one myofiber: wild‐type, *n* = 67; *Megf10^−/−^*, *n* = 71. The comparison of the cell counts between the wild‐type and *Meg10^−/−^* groups was performed by an unpaired *t*‐test; ***, *P* < 0.001. (C) The distances between Pax7169005038+/MyoD865006223+ satellite cells and their associated myofiber were measured at 72 h of culture. Each dot represents one Pax7+/MyoD+ cell. Arbitrary units noted on *y*‐axis. Wild‐type: *n* = 471 cells counted from 67 fibers; *Megf10^−/−^*: *n* = 165 cells counted from 71 fibers. The comparison of the migration distances between the wild‐type and *Megf10^−/−^* groups was performed by an unpaired *t*‐test; ***, *P* < 0.001. (D) MyoD+ nuclei are less abundant in *Megf10^−/−^* detached satellite cells compared to wild‐type cells. (E) Quantification of MyoD+ nuclei in proliferating satellite cells. *Megf10^−/−^* cells have significantly reduced MyoD+ nuclei in plated satellite cells after 96 h of single myofiber culture. Scatter plots represent counts from *n* = 10 images; mean ± SEM shown; ****, *P* < 0.0001.

To quantify satellite cell activation and migration patterns in greater detail, IF was performed to identify Pax7265947092+/MyoD619236543+ cells associated with individual myofibers, including those attached to and detached from the myofibers, at 72 h of culture (Fig. 2A). Manual counts of attached and detached Pax7+/MyoD+ cells showed that these cells were less abundant for *Megf10^−/−^* myofibers compared to wild‐type myofibers (Fig. 2B). Measurements of distances migrated by the Pax7+/MyoD+ cells at the same timepoint showed less robust migration for cells associated with *Megf10^−/−^* myofibers compared to wild‐type myofibers (Fig. 2C). Satellite cells detached from myofibers were cultured separately, showing less abundant MyoD expression among DAPI‐positive nuclei of *Megf10^−/−^* satellite cells compared to wild‐type satellite cells (Fig. 2D,E). These results provide additional evidence that Megf10 deficiency is associated with reduced satellite cell activation and migration.

### ***Reduced regeneration potential and increased fibrosis in Megf10*** *^−/−^* ***mice after muscle injury***

**Fig. 3:**
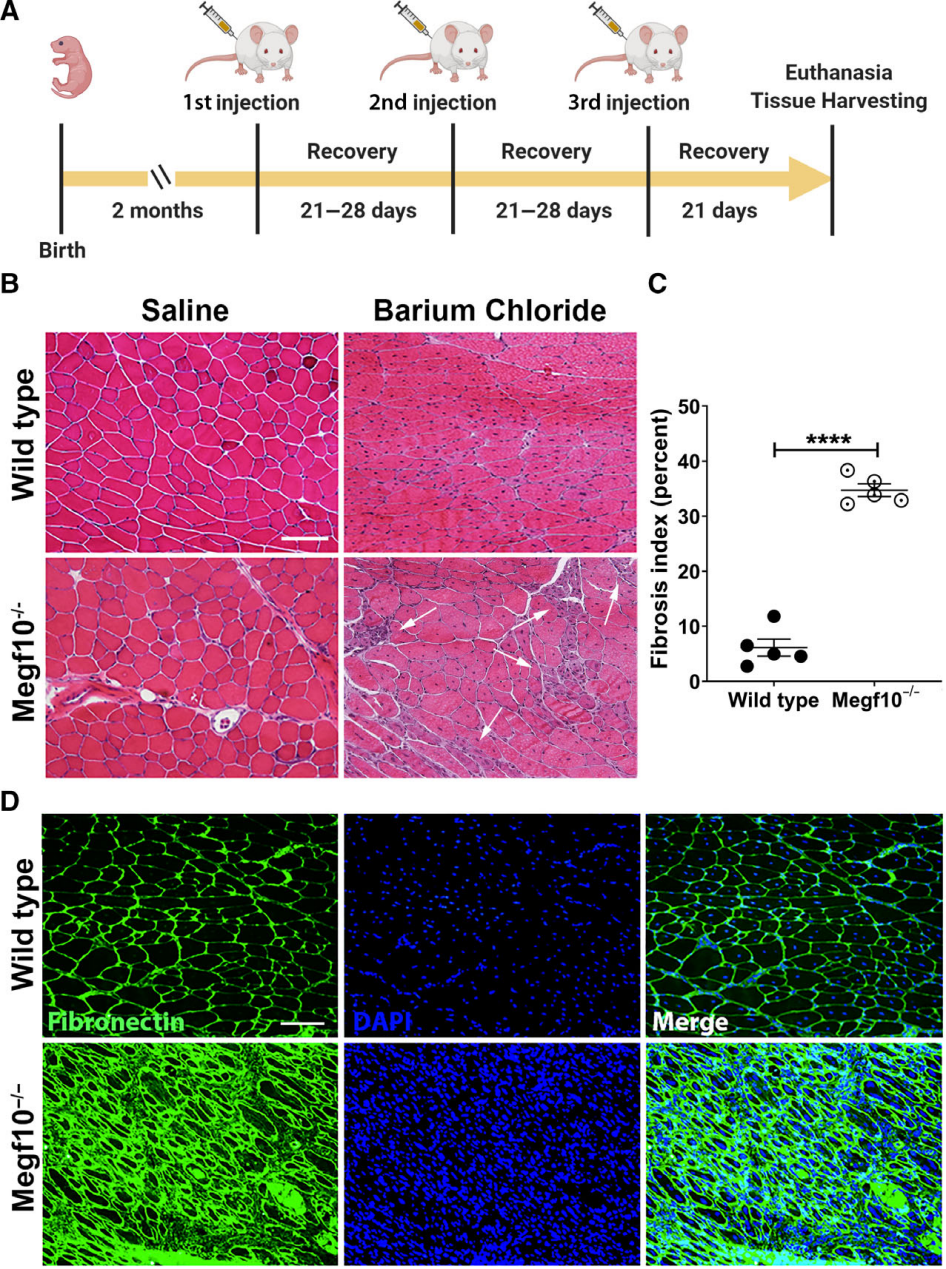
(A) Timeline of mouse injury experiments, showing intervals between birth, 3 sequential barium chloride and saline (control) injections into tibialis anterior, and euthanasia followed by tissue harvesting. (B) Hematoxylin and eosin staining of sections from tibialis anterior muscles that were injected with either barium chloride or saline in wild‐type and *Megf10^−/−^* mice. Arrows in the barium chloride *Megf10^−/−^* image indicate clusters of small myofibers with internalized nuclei; such clusters are not present in the barium chloride wild‐type image. Scale bar, 100μm. (C) Fibrosis quantification was performed on 5 images of fibronectin stained sections of tibialis anterior muscles injected with barium chloride in wild‐type and *Megf10^−/−^* mice. The percent fibrosis index was calculated as fibrotic area/total area × 100, indicated as percentages. An unpaired *t*‐test and one‐way ANOVA both yielded *P* < 0.0001 between wild‐type and *Megf10^−/−^* groups. Scatter plots represent fibrosis indices from *n* = 5 images; mean ± SEM shown; ****, *P* < 0.0001. (D) Representative IF images of muscle sections show fibronectin (green) with DAPI (blue) counterstaining in barium chloride injected tibialis anterior muscles from wild‐type and *Megf10^−/−^* mice. Scale bar, 100 μm.

Three sequential barium chloride injections into mouse tibialis anterior were performed for both wild‐type and *Megf10^−/−^* mice, with saline intramuscular injections serving as controls (Fig. 3A). Hematoxylin and eosin staining of representative sections from the injected muscles revealed significantly reduced regeneration potential with pronounced pockets of atrophic fibers and greater overall fiber size variability in *Megf10^−/−^* tibialis anterior muscles injected with barium chloride compared to wild‐type tibialis anterior muscles (Fig. 3B). Fibronectin staining was performed on muscle sections to demonstrate the degree of fibrosis in the injected tibialis anterior muscles. Greater densities of fibronectin were measured via ImageJ quantification in *Megf10^−/−^* muscles compared to wild‐type muscles (Fig. 3C). A representative IF image demonstrates the thicker bands of fibronectin seen in *Meg10^−/−^* muscle sections compared to wild‐type muscle sections (Fig. 3D).

## Discussion

In the decade since the gene *MEGF10* was first associated with a distinct human muscle disease, it has become apparent that the protein product MEGF10 plays a key role in satellite cell function. Over time, various aspects of MEGF10’s biochemical and cellular functions have been elucidated. The current study fills in another piece to the puzzle and provides further details on how MEGF10 deficiency affects satellite cell behavior and offers novel information regarding the impact of such a deficiency on skeletal muscle regenerative capacity.

Satellite cells are mononuclear progenitor stem cells in skeletal muscle that make key contributions to the process of muscle repair after injury. The name ‘satellite cell’ was derived from the original observation that these cells are found in the niche between the basal lamina and sarcolemma of a myofiber [12]. The satellite cell cycle includes symmetric and asymmetric components, the former consisting of self‐renewal within the quiescent state [29] and the latter leading to a terminal exit from that state. Quiescent and self‐renewing satellite cells can be identified by the detection of several distinct protein markers, most commonly the paired box 7 (Pax7) transcription factor in their nuclei [30]. Of particular relevance to Megf10, which interacts with Notch1 [10, 11], extrinsic Notch signaling is critical for the maintenance of the quiescent state [31, 32] and Notch1 activation promotes proliferation of satellite cells [14]. The classic stages of an exit from the quiescent state are demarcated by the expression patterns of key myogenic regulatory factors (MRFs) that are all basic helix‐loop‐helix (bHLH) transcription factors [33]: Myf5 [34], MyoD [35], myogenenin [36], and Mrf4 [37]. All four of these MRFs are induced during various stages of satellite cell activation [38, 39]. For our studies, we focused on Pax7+/MyoD+ cells as the co‐expression of these two markers is generally accepted as indicating the presence of activated satellite cells that proliferate and migrate [40], and our prior work indicated that Megf10 deficiency is associated with impaired proliferation and migration in C2C12 myoblasts and primary mouse myoblasts [11], suggesting a role for Megf10 in this early stage of the satellite cell myogenic pathway.

In this context, what role might MEGF10 play in the regulation of the early stages of the satellite cell myogenic pathway? It is an orphan receptor that is expressed at the cell surface with a single transmembrane domain and thus is not an MRF. It interacts with Notch1 and thus likely performs an extrinsic regulatory task [10], though the interaction occurs at both proteins’ intracellular domains [11]. Our current data show that Megf10 deficiency impairs migration of Pax7+/MyoD+ activated satellite cells and is associated with reduced numbers of MyoD + cells, providing further evidence that Megf10 contributes to an early stage of satellite cell activation during which the cells migrate to target myofibers. This information could help explain the persistent nature of the clinical manifestations of MEGF10 myopathy, as muscle function generally does not improve in human patients affected by this disease [1, 2].

In previous studies, we have shown that downregulation of *draper (drpr)*, the Drosophila homolog of *Megf10*, in quiescent adult muscle precursors (satellite cell‐like AMPs) leads to decreased motor activity in corresponding mutant flies [41]. These cells, however, are insensitive to *drpr/Megf10* overexpression, which is deleterious later in myogenesis, that is, at the migration/differentiation stages (suggesting that Drpr levels are finely tuned during these steps) [42]. It is well established that *drpr* plays a crucial role in mediating the migration of glia toward injured neurons [43, 44], as well as the migration of immune cells toward tissue wound [45]. Whether a parallel *drpr/Megf10*‐dependent molecular mechanism participates in regulating the migration of satellite cells toward developing, or injured, muscle fibers remains to be elucidated. Regeneration of injured muscle has been reported in Drosophila [46]. The fly may provide a useful model in which to further elucidate the conserved pathways downstream of *drpr/Megf10* that contribute to muscle development and potentially repair. Future Drosophila studies could address the question of satellite cell migration by examining the effects of *drpr* deficiency on the migration patterns of AMPs, which behave much as muscle satellite cells do in mammals [47].

Regardless of the exact cellular role of Megf10 in the satellite cell cycle, our data show a clear impact on skeletal muscle regeneration. Impaired muscle regeneration has significant chronic consequences for muscle homeostasis. Thus, MEGF10 bears further investigation as a therapeutic target, not only for MEGF10 deficiency itself, but also for other inherited muscle diseases, including various forms of muscular dystrophy. Recently, we published a study that identified selective serotonin reuptake inhibitors, notably sertraline, as having a beneficial effect on cellular and *in vivo* (i.e., Drosophila and zebrafish) models of Megf10 deficiency [48]. Serotonin did not replicate this effect in Drosophila, whereas sertraline behaved as a Notch pathway agonist in these cellular models, suggesting another link between MEGF10 and the Notch pathway [48] in addition to evidence from various selective serotonin reuptake inhibitors (SSRIs) in other contexts [49, 50, 51, 52]. Further investigations of the effects of sertraline on Megf10 deficiency should include detailed mouse experiments that could both confirm the therapeutic effect and also elucidate the interactions between Megf10 and the satellite cell cycle. These studies could then be supplemented by examinations of the effects of sertraline on mouse models for other inherited muscle diseases, to explore potential broader applications of manipulating this therapeutic target.

Overall, more precise information regarding the relationship between MEGF10 and the Notch pathway could be drawn out by exploring potential interactions with other Notch pathway components, as well as with protein O‐glucosyltransferase 1 (encoded by *POGLUT1* and previously known as Rumi); the latter is associated with muscular dystrophy and is also known to be a regulator of the Notch pathway [53, 54]. *POGLUT1* mutations have been associated with a reduction in muscle satellite cells [55]. Detailed fluorescence activated cell sort (FACS) experiments could also elucidate how MEGF10 deficiency could alter early stages of the satellite cell cycle. Such knowledge could then lead to the development of standardized *in vitro* markers to gauge potential therapeutic effects of sertraline or other candidate therapies for MEGF10 myopathy. MEGF10 could provide a therapeutic target for manipulation of Notch signaling to augment skeletal muscle regenerative capacity via satellite cell enhancement, which could lead to new treatments not only for MEGF10 myopathy, but also for other inherited muscle diseases accompanied by impaired muscle regeneration, including a broad range of muscular dystrophies.

## Conflict of interest

The authors declare no conflict of interest.

## Author contribution

PBK conceived and supervised the study and designed experiments. CL, MS, RMD, and KAM performed experiments. CL, DV, MS, CAP, and PBK analyzed data. CL, DV, MS, ID, CAP, and PBK interpreted data and wrote the manuscript.

## Data Availability

The corresponding author will share data upon reasonable request.
